# Proctitis Caused by *Mycobacterium avium-intracellulare* in an HIV-Infected Patient

**DOI:** 10.3390/diseases6020036

**Published:** 2018-05-08

**Authors:** Jose Armando Gonzales Zamora, Clara Milikowski

**Affiliations:** 1 Division of Infectious Diseases, Department of Medicine, Miller School of Medicine, University of Miami, Miami, FL 33136, USA; 2 Division of Anatomic Pathology, Department of Pathology and Laboratory Medicine, University of Miami, Miami, FL 33136, USA; CMilikowski@med.miami.edu

**Keywords:** proctitis, *Mycobacterium avium-intracellulare*, HIV

## Abstract

Infectious proctitis is usually associated with sexually transmitted diseases, especially in HIV-infected individuals. Limited information is found about the role of *Mycobacterium avium-intracellulare* as a causative agent for this condition. Here, we report the case of an HIV-infected patient with a CD4 count of 304 cells/uL and undetectable HIV viral load, who presented with constipation and painful defecation. Endoscopic evaluation was significant for shallow rectal ulcerations. Histopathology revealed poorly formed granulomas. Stool culture grew *Mycobacterium* sp. that was further identified as *Mycobacterium avium-intracellulare* by DNA probe. He was successfully treated with a 3-drug regimen that included azithromycin, ethambutol and rifabutin. We advocate the use of AFB stool culture in cases of proctitis in which initial investigations for sexually transmitted diseases are unrevealing.

## 1. Introduction

Proctitis is an inflammation of the rectal mucosa, and is frequently encountered in HIV-infected men who have sex with men (MSM). In this population, sexually transmitted diseases constitute the leading cause of infectious proctitis. The most common etiologic agents are *Herpes simplex*, *Chlamydia trachomatis* and *Neisseria gonorrhoeae* among others; however, a specific cause cannot be identified in a high number of cases [1]. *Mycobacterium avium-intracellulare* (MAI) is an environmental pathogen that has not been recognized as a causative agent of proctitis. This non-tuberculous *Mycobacterium* is commonly responsible for disseminated disease in severely immunocompromised HIV patients with CD4 counts lower than 50 cells/uL. Gastrointestinal involvement has been documented in several reports, with the duodenum, being the most commonly affected organ [2]. Here, we describe the case of an HIV-infected patient with localized proctitis secondary to *Mycobacterium avium-intracellulare* that was successfully treated with a three-drug regimen. Rectal involvement by MAI has been reported only as part of a disseminated process affecting the colon or other extraintestinal organs. To the best of our knowledge, the present case is the first description of MAI infection exclusively affecting the rectum. In this report, we also discuss the challenges we usually face for the diagnosis and management of this infection.

## 2. Case Report

**Figure 1:**
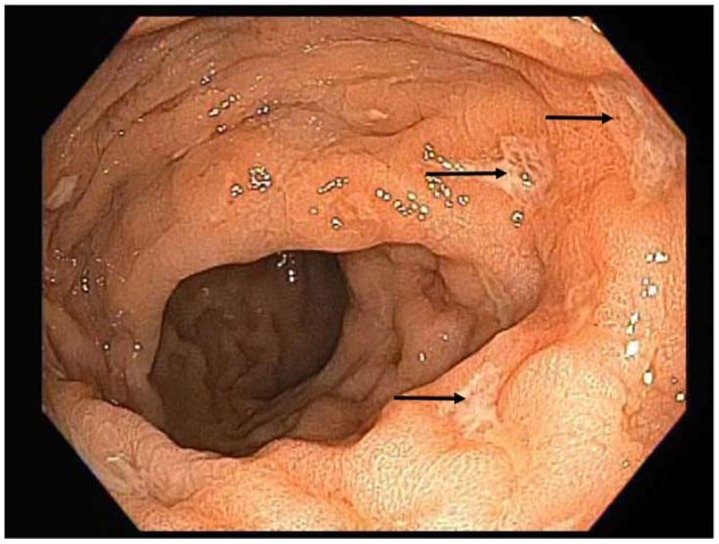
Colonoscopy showing shallow ulcerations in rectal mucosa (arrow).

**Figure 2:**
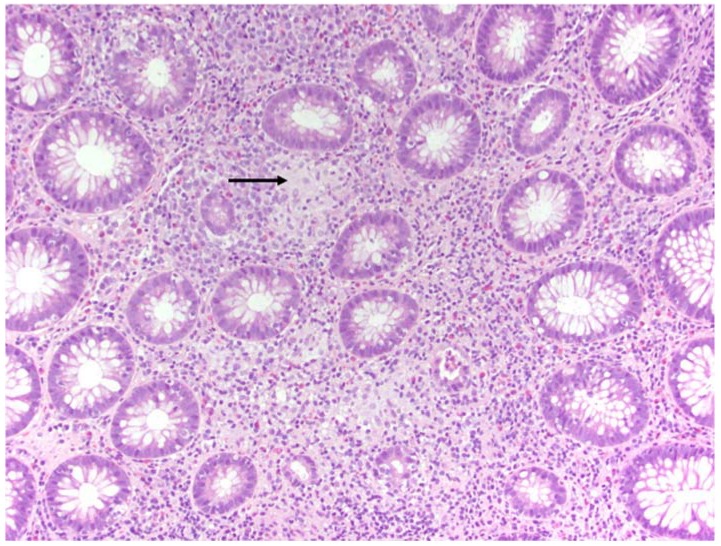
Histopathology showing cryptitis and noncaseating granuloma (arrow) in rectal mucosa (H&E, 400×).

A 48-year-old man originally from Mexico presented to the Infectious Disease clinic with constipation and painful defecation in the last 4 weeks. He had noticed streaks of blood on the toilet paper after wiping. He had a 5-year history of HIV infection treated initially with Tenofovir/emtricitabine/efavirenz that was recently switched to abacavir/lamivudine/dolutegravir. His latest CD4 count was 304 cells/uL and his HIV viral load was undetectable. He was MSM (men who have sex with men), but denied unprotected sex in the last 3 months. His vital signs were within normal limits. He weighed 77.1 kg and did not report any significant weight loss. His abdomen was soft, nontender and non-distended. Bowel sounds were normoactive. Rectal exam was normal. Laboratory studies showed hemoglobin of 14.1 g/dL, leukocyte count of 4.7 K cells/uL and platelet count of 246 K cells/uL. Chemistry panel was significant only for mildly elevated alkaline phosphatase (142 U/L). RPR (rapid plasma reagin) was negative. Stool culture was negative for *Salmonella*, *Shigella*, *Aeromona* and *Plesiomona*. *Campylobacte*r antigen and *Escherichia coli* shigatoxins were not detected. Ova and parasites were not isolated in stool studies. He underwent rectal swab for gonorrhea and chlamydia PCR, which gave negative results. Given unrevealing work-up, the patient was referred to Gastroenterology for endoscopic evaluation. Colonoscopy showed moderate inflammation characterized by congestion, erythema and friability of the rectal mucosa. Shallow ulcerations were noted only in the rectum (Figure 1). The rest of the colon did not show any abnormalities. Histopathology examination of rectal tissue disclosed a mucosa with increased lymphoplasmacytic and neutrophilic infiltrate in lamina propia. Acute cryptitis and focal crypt abscesses were noted, along with erosions and few poorly formed granulomas (Figure 2). Immunohistochemistry for HSV1, HSV2, and CMV was negative. Tissue AFB (acid-fast bacilli) staining did not show any organisms. AFB smear in stool was negative; however, two weeks later, stool culture grew *Mycobacterium* sp. that was further identified as *Mycobacterium avium-intracellulare* complex by DNA probe. No susceptibility testing was performed. Rectal tissue culture did not grow any organisms. To complete the work-up, a chest X-ray and quantiferon gold were ordered. The results were unremarkable. The patient was started on azithromycin 500 mg daily, rifabutin 300 mg daily and ethambutol 1200 mg daily. At 2-month follow up, the patient reported complete resolution of his symptoms. Four months later, rifabutin was discontinued. The plan was to continue with azithromycin and ethambutol for six additional months to complete a total treatment course of one year.

## 3. Discussion

Proctitis is an inflammation of the rectal mucosa and is restricted to the distal 15 cm of the colon. Symptoms vary depending on the specific pathological process. The most common symptom is a continuous urge to have a bowel movement. Other symptoms include anorectal pain, anal discharge, constipation and rectal bleeding [3]. This condition has several non-infectious and infectious causes. In cases of infectious proctitis, the cause is usually a sexually transmitted disease. In a retrospective study conducted by Klausner et al. in San Francisco, the most common pathogens were gonorrhea (30%), chlamydia (19%), HSV-2 (16%) and syphilis (2%). No identifiable infectious source was found in 46% of cases. Co-infections were not uncommon, 10% of the patients with infectious proctitis tested positive for multiple pathogens [4].

The HIV status is a factor that seems to influence the etiological distribution of pathogens associated with proctitis. Bissessor et al. conducted a study in Australia that aimed to compare the spectrum of microorganisms responsible for infectious proctitis between HIV-infected and HIV-non-infected men who have sex with men. They found that, compared with the rest of the study cohort, HIV-infected individuals had a higher rate of proctitis caused by lymphogranuloma venereum (7.8% vs. 0.7%, *p* = 0.004), HSV-1 (14.2% vs. 6.5%, *p* = 0.04), HSV-2 (22% vs. 12.3%, *p* = 0.03) and multiple infections (17.7% vs. 8.6%, *p* = 0.017). Of note, 28% of HIV-infected individuals did not have any pathogen detected, which was significantly lower that the percentage observed in HIV-negative patients [5].

Our patient presented classic symptoms of proctitis; however, appropriate investigations for sexually transmitted infections were unrevealing. Further work-up disclosed the diagnosis of MAI proctitis. This non-tuberculous *Mycobacterium* has been recognized as one of the most common etiologies of opportunistic infections in patients with acquired immunodeficiency syndrome (AIDS). MAI is ubiquitous in the environment and the mode of infection seems to be through inhalation or ingestion. In homosexual men, venereal transmission has also been suggested [6]. In our patient, there was no evidence of pulmonary involvement and he denied any recent sexual contact. We believe that rectal seeding may have occurred via hematogenous spread or by ingestion.

The risk of MAI infection in patients with HIV increases as the CD4 count drops below 50 cells/uL. One of the unique characteristics of our patient is his relatively high CD4 count (304 cells/uL) on presentation, which is uncommon in HIV patients with MAI infection. There may be a genetic predisposition for acquiring this infection. In a case-control study from the Multicenter AIDS Cohort, specific human leukocyte antigen (HLA) class II alleles (DRB1, DQB, DM) were more commonly associated with MAI disease [7]. An innate susceptibility could explain the development of MAI infection in our patient.

The two main manifestations of MAI infection in HIV-infected patients are disseminated and localized disease. Disseminated disease was the predominant form prior to the widespread use of antiretroviral therapy. The symptoms of disseminated MAI are nonspecific and include fever, night sweats, diarrhea and abdominal pain. Gastrointestinal symptoms are reported in approximately 40% of disseminated cases and they include diarrhea, malabsorption and gastrointestinal hemorrhage [8,9,10]. Within the gastrointestinal tract, the duodenum is the most commonly involved organ. The case series published by Gray et al. describes duodenal involvement in 88% of cases of gastrointestinal MAI infection. Other affected organs were the esophagus and the liver. Rectal involvement has also been described, but as part of a disseminated process affecting the colon and other extra-intestinal areas [2,11]. In our patient, only the rectal mucosa was involved, which constitutes a presentation never reported before.

The diagnosis of MAI infection typically relies on cultures. In cases of disseminated infection, blood culture is the initial preferred diagnostic test, given its high sensitivity and lower invasiveness. In this scenario, there is a limited role for obtaining stool cultures. A prospective study found that colonization of the gastrointestinal and respiratory tract had a sensitivity of only 20% and a positive predictive value of 60% for detecting disseminated disease [12]. On the other hand, in cases of proven gastrointestinal involvement, stool culture has demonstrated to be a valuable test, with an overall sensitivity as high as 86% and a specificity of 99% [2,13,14]. Tissue culture has shown lower detection rates (76%). The histopathology of MAI infection is usually characterized by noncaseating granulomas resembling Crohn’s disease. Small bowel biopsies can also show severe villous flattening with aggregates of foamy macrophages full of intracellular acid-fast organisms [15,16]. One of the distinctive endoscopic features noted in cases of duodenal MAI infection is the presence of diffusely whitish nodules, found in approximately 35% of cases [2,17]. Mucosal friability, superficial ulceration and diffuse erythema are endoscopic characteristics described in cases of MAI colitis [10]. In our patient, the diagnosis was achieved by stool culture and histology; and it was subsequently confirmed by adequate response to anti-mycobacterial treatment.

In patients with AIDS, combination antimicrobial therapy is recommended for treatment of MAI infection. Dual therapy with macrolides and ethambutol is the cornerstone of MAI treatment. A third agent is usually added in cases of advanced immunosuppression, high mycobacterial loads, or absence of effective antiretroviral therapy [18]. Rifabutin is the preferred third drug, but other alternatives include levofloxacin, moxifloxacin or amikacin. Treatment should be continued for at least 12 months; however, the ultimate duration depends upon how fast the patient recovers his immunologic function after initiating antiretroviral therapy, since the CD4 count should be stably above 100 cells/uL for at least six months before discontinuing treatment [18]. In HIV-non-infected patients, the duration of therapy depends on the organ involved. In cases of pulmonary MAI infection, the treatment should be continued until sputum cultures are consecutively negative for 12 months. Since sputum conversion usually takes 3 to 6 months of treatment, a typical patient is treated for 15 to 18 months. In cases of extrapulmonary MAI infection, the optimal duration of treatment has not been well established, and treatment is habitually administered for at least 6 months [19]. We decided to treat our patient for 1 year given his immunocompromised state; however, he was not in late stages of HIV infection, as are most of the patients included in studies of disseminated MAI. This could raise the point that a shorter course of treatment could have probably been equally effective in our patient. Further studies are needed to determine the optimal duration of treatment in cases of localized extrapulmonary MAI infection, especially when CD4 counts are above 200 cells/uL.

## 4. Conclusions

Proctitis secondary to MAI is very uncommon, even in the setting of HIV infection. We believe that stool AFB and culture should be part of the work-up in patients presenting with proctitis, in whom initial investigations for sexually transmitted diseases are negative. The treatment for MAI is typically based on a 3-drug regimen that includes macrolides, ethambutol and possibly rifabutin. The optimal duration of therapy for extrapulmonary disease has not been standardized; however, a six- to twelve-month course is usually advocated.
